# Lack of clinically relevant correlation between subjective and objective cognitive function in ICU survivors: a prospective 12-month follow-up study

**DOI:** 10.1186/s13054-019-2527-1

**Published:** 2019-07-12

**Authors:** Emily Brück, Jacob W. Larsson, Julie Lasselin, Matteo Bottai, Tatja Hirvikoski, Eva Sundman, Michael Eberhardson, Peter Sackey, Peder S. Olofsson

**Affiliations:** 1 Function Perioperative Medicine and Intensive Care, Karolinska University Hospital Solna, 171 76 Stockholm, Sweden; 2 Laboratory of Immunobiology, Center for Bioelectronic Medicine, Department of Medicine, Karolinska Institutet, 171 77 Stockholm, Sweden; 3 Department of Physiology and Pharmacology, Karolinska Institutet, 171 77 Stockholm, Sweden; 4 Stress Research Institute, Stockholm University, 106 91 Stockholm, Sweden; 5 Division of Psychology, Department of Clinical Neuroscience, Karolinska Institutet, 171 77 Stockholm, Sweden; 6 The Unit of Biostatistics, Institute of Environmental Medicine, Karolinska Institutet, 17177 Stockholm, Sweden; 7 Department of Women’s and Children’s Health, KIND, Karolinska Institutet, Karolinska University Hospital, 171 76 Stockholm, Sweden; 8 REMEO Stockholm, Torsten Levenstams väg 4, 128 64 Sköndal, Sweden; 9 Center for Biomedical Science, The Feinstein Institute for Medical Research, Manhasset, New York, 11030 USA

**Keywords:** Critical care, Intensive care unit, Cognitive impairment, Post-traumatic stress symptoms, Anxiety, Depression, CANTAB, CFQ

## Abstract

**Background:**

Cognitive impairment and psychological distress are common in intensive care unit (ICU) survivors. Early identification of affected individuals is important, so intervention and treatment can be utilized at an early stage. Cognitive Failures Questionnaire (CFQ) is commonly used to screen for subjective cognitive function, but it is unclear whether CFQ scores correlate to objective cognitive function in this population.

**Methods:**

Between 2014 and 2018, 100 ICU survivors aged 18–70 years from the general ICU at the Karolinska University Hospital, Solna, were included in the study. Out of these, 58 patients completed follow-up at 3 months after ICU discharge, 51 at 6 months, and 45 at 12 months. Follow-up included objective cognitive function testing using the Cambridge Neuropsychological Test Automated Battery (CANTAB) and subjective cognitive function testing with the self-rating Cognitive Failures Questionnaire (CFQ), as well as psychological self-rating with the Post-Traumatic Stress Symptoms Scale-10 (PTSS-10) and Hospital Anxiety and Depression Scale (HADS).

**Results:**

The prevalence of cognitive impairment as measured by four selected CANTAB tests was 34% at 3 months after discharge, 18% at 6 months, and 16% at 12 months. There was a lack of significant correlation between CANTAB scores and CFQ scores at 3 months (*r* = − 0.134–0.207, *p* > 0.05), at 6 months (*r* = − 0.106–0.257, *p* > 0.05), and at 12 months after discharge (*r* = − 0.070–0.109, *p* > 0.05). Correlations between CFQ and PTSS-10 scores and HADS scores, respectively, were significant over the follow-up period (*r* = 0.372–0.710, *p* ≤ 0.001–0.023). In contrast, CANTAB test scores showed a weak correlation with PTSS-10 and HADS scores, respectively, at 3 months only (*r* = − 0.319–0.348, *p* = 0.008–0.015).

**Conclusion:**

We found no clinically relevant correlation between subjective and objective cognitive function in this cohort of ICU survivors, while subjective cognitive function correlated significantly with psychological symptoms throughout the follow-up period. Treatment and evaluation of ICU survivors’ recovery need to consider both subjective and objective aspects of cognitive impairment, and subjective reports must be interpreted with caution as an indicator of objective cognitive function.

## Background

Every year, several million patients are admitted to intensive care units (ICUs) in Europe due to unexpected and life-threatening illness or injury [1], and the majority of patients survive the acute episode [2]. However, among these ICU survivors, one in three [3, 4] suffer from post-intensive care syndrome (PICS) [5]. PICS includes cognitive impairment, depression, post-traumatic stress disorder, and functional disabilities, which result in a marked reduction of quality of life in the months to years after ICU discharge [3, 4, 6–9]. PICS, including cognitive impairment, has become recognized as an important public health problem, as the long-term consequences of PICS may prevent ICU survivors from returning to the level of functioning they had before the onset of critical illness [10]. Cognitive functions commonly impaired after intensive care include memory, attention, processing speed, visuospatial ability, and executive function [11]. It has been shown that PICS symptoms are related, and we have previously found that cognitive problems in ICU survivors are associated with symptoms of increased post-traumatic stress, anxiety, and depression [12]. The reported prevalence of cognitive impairment as measured by neuropsychological tests in ICU survivors is 4–62% [7, 11, 13]. This large range might be due to that cognitive testing post ICU differs in follow-up time and conducted tests [13].

Objective measurements of cognitive function including neuropsychological test batteries used in ICU follow-up research are expensive, time consuming, and labor intensive. A simpler screening tool that identifies patients with cognitive impairment for further evaluation and support would be very helpful. In ICU survivors, Cognitive Failures Questionnaire (CFQ) has been used to assess cognitive impairment by patient self-rating [6, 14, 15]. However, self-rating questionnaires often fail to detect impaired cognition [16]. CFQ was designed to measure patients’ perception of cognitive difficulties in daily life [17], and it is not known whether CFQ scores are suitable for identifying patients with cognitive impairment among ICU survivors. Observations from other patient populations are conflicting on the correlation of CFQ scores and objective measurements of cognitive function [18, 19]. Accordingly, it is important to investigate whether CFQ scores reflect impaired cognition in ICU survivors. Furthermore, it is not known whether objective cognitive impairment correlates to signs of post-traumatic stress, anxiety, and depression in this population.

The primary aim of this study was to investigate whether there is a clinically relevant correlation in ICU survivors between subjective cognitive function measured by the CFQ and objective cognitive function measured by four selected CANTAB tests assessing executive function, working memory, visual memory, and attention. In addition, we studied whether depression, anxiety, and post-traumatic stress symptoms in ICU survivors correlate to cognitive impairment as measured by the CFQ and CANTAB, respectively.

## Methods

### Study design

We conducted a prospective observational cohort study at the Karolinska University Hospital, Solna, Sweden, in which patients were followed up to 12 months after ICU discharge. The study was approved by the Regional Ethical Review Board in Stockholm (approval number 2013/1221-31/1).

### Patients

Patients aged 18–70 admitted to the general ICU at the Karolinska University Hospital in Stockholm for more than 24 h were eligible for inclusion. Exclusion criteria included serious auditory or visual disorders, aphasia, or inability to understand Swedish. Patients diagnosed with mental impairment, including dementia, were also excluded. All patients above 50 years of age were screened by research nurses at inclusion (at the ICU) for early cognitive decline with the Short Informant Questionnaire on Cognitive Decline in the Elderly (IQCODE, 1.0–5.0), and patients scoring > 3 were excluded [20]. Patients with an expected ICU stay of less than 48 h, patients transferred to other ICUs, and patients residing outside Stockholm County were also excluded. Patients with alcohol or drug abuse, diagnosed with an ongoing psychiatric illness, or having a psychiatric pharmacological treatment were excluded. Furthermore, patients with meningitis, with structural brain injury, on extracorporeal membrane oxygenation (ECMO), in palliative care, or deemed unlikely to survive to follow-up (3 months) were excluded.

### Data collection

Data were collected from May 2014 to January 2018. Patients were not included in June–August and during holidays when research nurses were unavailable. Patient characteristics (age, comorbidities) and ICU-related information (mechanical ventilation, SAPS III, APACHE II, sepsis status, presence of delirium) were collected from the electronic patient data management system and through medical chart review.

Patients were followed up with clinical assessments at 3, 6, and 12 months after ICU discharge coming to the outpatient clinic for Intensive Care at the Karolinska University Hospital, Solna. They performed conventional neuropsychological tests (CANTAB) and handed in three self-rating questionnaires (CFQ, PTSS-10, and HADS) that were sent to the patient by regular mail two weeks before each visit.

### Assessment of subjective cognitive function

The Cognitive Failures Questionnaire (CFQ) was used to assess subjective cognitive function. It is a self-rating questionnaire consisting of 25 questions that concern the frequency of certain cognitive difficulties, with a total score of 0–100. Higher scores indicate more subjective cognitive difficulties. The CFQ covers four dimensions of cognition (memory, e.g., “Do you find you forget appointments?”; distraction, e.g., “Do you read something and find you haven’t been thinking about it and must read it again?”; social blunders, e.g., “Do you lose your temper and regret it”; and naming, e.g., “Do you find you can’t quite remember something although it’s ‘on the tip of your tongue’?”) [17, 21]. CFQ is validated in ICU settings [14].

### Assessment of objective cognitive function

Objective cognitive function was measured using the Cambridge Neuropsychological Test Automated Battery (CANTAB® [Cognitive assessment software], Cambridge Cognition (2013)). CANTAB is a touchscreen computer-based neuropsychological test battery that is non-verbal and is validated for repeated testing.

A 30-min test battery was administered in a silent room to minimize disturbances. Standard protocols provided by the test developer were used, and a trained research assistant conducted the procedure.

At the beginning of the test, the motor screening (MOT) test was used as an introduction to the touch screen, and a general assessment of visual or motor impairment that could affect the other cognitive tests. In the MOT, X marks in different locations and colors appear on the screen, and the subject has to touch them as quickly as possible. All patients’ successfully completed MOT right before the other tests were conducted.

We used four tests from CANTAB to measure executive function, working memory, visual memory, and attention.

#### Stockings of Cambridge (SOC)

The SOC tests executive function, in particular spatial planning and spatial working memory, and is a computerized version of the Tower of London Task [22]. Colored circles are arranged in stacks, and the subject must move the circles to match a template. The difficulty rises with the number of moves required to match the template (from two to five moves). Outcomes of the SOC are the average number of moves needed to solve each task (for each level of difficulty) and the number of problems the subject can solve with the minimum number of moves requested (“SOC—problems with min moves”). We used the latter as an overall assessment of SOC performance.

#### Pattern recognition memory (PRM)

The PRM tests visual memory. A series of 12 different geometric patterns are shown in the middle of the screen, one at a time. The subject is instructed to remember the patterns. After the initial series, the recognition phase takes place, in which the subject is shown two patterns, one previously presented and a novel one. The subject is asked to indicate the pattern he/she recognizes. The outcome of the PRM is the percentage of correct responses (“PRM—% correct”).

#### Spatial span (SSP)

The SSP assess visual-spatial and working memory and is based on the Corsi Blocks Task [23]. Ten white boxes are presented on the screen, and the color of the boxes changes one by one in an order that the subject must remember. The subject is then to reproduce the same sequence by touching the boxes. Level of difficulty increases with the number of boxes the subject has to remember (from two to nine). The outcome of the SSP is the length of the maximum sequence the subject could remember (“SSP—span length”).

#### Rapid visual information processing (RVP)

The RVP tests sustained attention and is similar to the Continuous Performance Task [24]. A white box is shown at the center of the screen, where digits from 2 to 9 appear in a random order, at the rate of 100 digits per minute. The subject is instructed to touch the pad button as quickly as possible every time they detect certain sequences (i.e., 2–4–6, 3–5–7, or 4–6–8). Outcomes of the RVP are the average and median latency to answer, the probability of hit, the total number of false alarms, and a sensitivity score, which reflects how well the subject can detect the target sequences (range 0.00 to 1.00; bad to good) (“RVP A’”). The latter was used as an overall estimation of performance in the RVP.

These four tests rendered twelve outcome measures. For eleven of the twelve outcome measures, a *z*-score was provided by the CANTAB software, which was derived from an age- and gender-matched British norm population mean. The presence of cognitive impairment was defined as having two out of eleven *z*-scores below − 2.0 or three out of eleven *z*-scores below − 1.5, as suggested in previous studies [25, 26]. To measure objective cognitive function, we selected one outcome for each test to minimize multiple comparisons.

### Assessment of post-traumatic stress, anxiety, and depression

Symptoms of post-traumatic stress, anxiety, and depression were measured using two self-rating questionnaires:

a) Post-Traumatic Stress Symptoms Scale-10 (PTSS-10), which assesses PTSD-related symptoms by ten questions related to current post-traumatic stress symptoms [27]. The symptoms are graded from 1 (“never”) to 7 (“always”), with a maximum score of 70. The questionnaire is reportedly reliable for assessing post-traumatic stress symptoms in former ICU patients [21, 27].

b) Hospital Anxiety and Depression Scale (HADS), which consists of two subscales that measure symptoms of anxiety and depression with a maximum subscale score of 21. HADS has been validated for detecting symptoms of anxiety and depression in ICU patients [28–30].

### Statistical methods

Numeric demographic variables were summarized with medians and interquartile ranges. Absolute and relative frequencies were reported for categorical variables. The mean values of CFQ and the four CANTAB tests over time were estimated with linear random-intercept models. Separately for each time point, we calculated the Spearman’s rank correlation coefficient between CFQ and the four CANTAB tests, and CFQ and HADS or PTSS-10, respectively, using the raw score of PRM—% correct, SOC—problems with min moves, RVP A’, SSP—span length, and the CFQ sum score. *p* values less than 0.05 were considered significant. STATA version 15 (StataCorp, College Station, TX, USA) was used for the analyses.

## Results

**Fig. 1:**
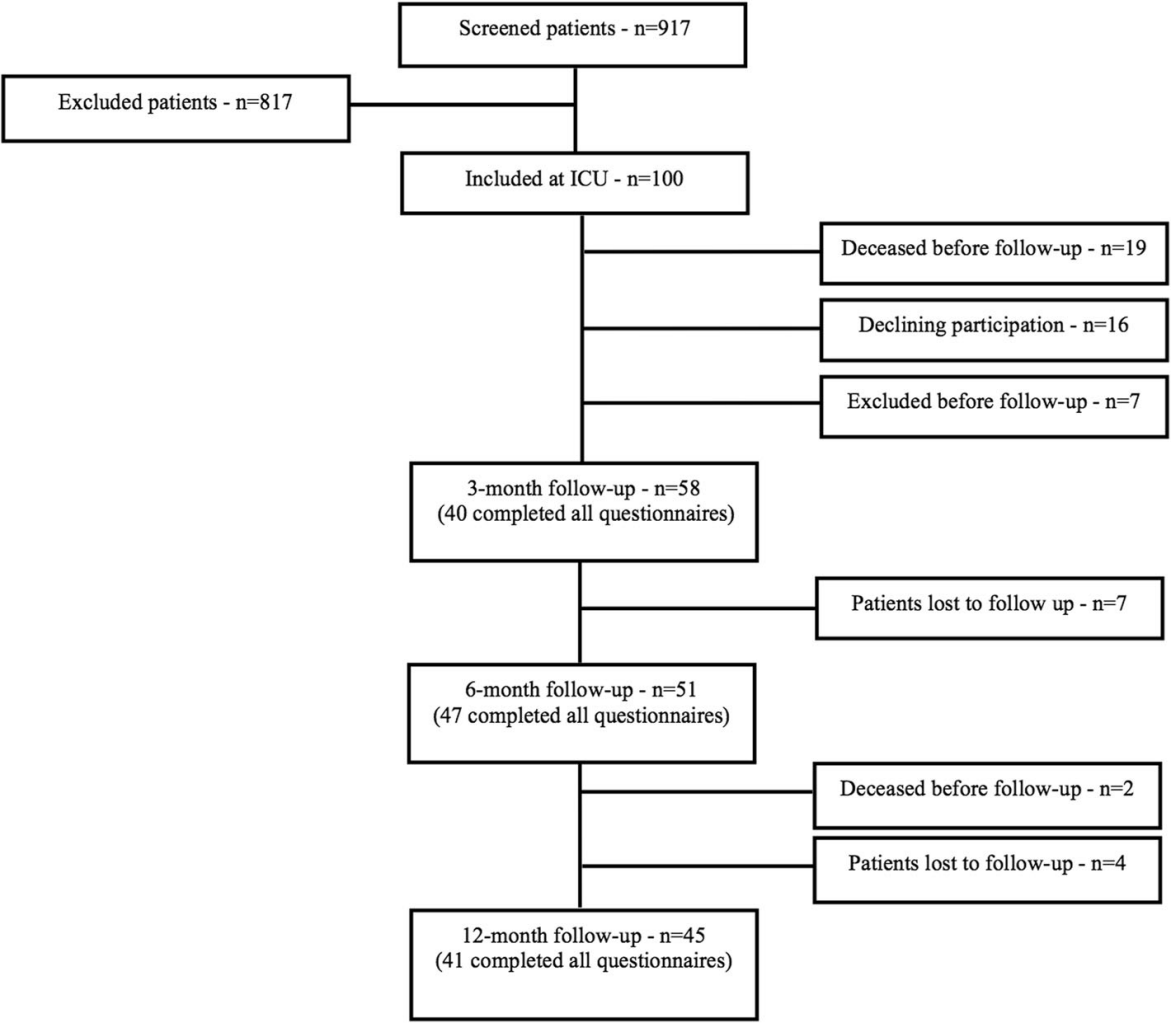
Flow chart of patient inclusion in the study

Between 2014 and 2018, 917 patients admitted to the general ICU at the Karolinska University Hospital were screened for eligibility (aged 18–70, staying more than 24 h) and 100 of these patients met the inclusion criteria (see Table 1 for information on excluded patients). Of these 100 patients, 58 patients performed CANTAB at the 3-month follow-up, among them 40 patients also completed the CFQ. Corresponding numbers for follow-up at 6 months were 51 and 47, and finally 45 and 41 patients at 12 months (Fig. 1). Patient characteristics at the first follow-up time point (i.e., at 3 months) are presented in Table 2.

**Table 1:** Exclusion criteria

1. Does not speak/understand Swedish	60
2. Abuse of alcohol or drugs	127
3. Aphasia, blindness, or deafness	10
4. Ongoing psychiatric disorder or psychopharmacological drug treatment	45
5. Dementia, ongoing screening for dementia or cognitive deficit	27
6. Structural brain damage or meningitis	181
7. Treatment limitations or palliative care	38
8. ICU treatment < 48 h	47
9. Patient received from other ICU units	123
10. ECMO	8
11. Out-of-county patient	61
12. Other reasons	90
No. of excluded in total	817

**Table 2:** Demographic and clinical characteristics at 3-month follow-up

	Follow-up cohort at 3 months (*N* = 58)
Age, year, median (IQR)	54 (41–64)
Male sex, no. (%)	44 (76)
Level of education, no. (%)	
Primary	15 (26)
Secondary	24 (41)
Tertiary	19 (33)
Nicotine abuse, no. (%)	22 (38)
Comorbidity, no. (%)	
Cardiovascular	18 (31)
Respiratory	7 (12)
Gastrointestinal	3 (5)
Diabetes	6 (10)
Cancer	8 (14)
Immunological	2 (3)
Neurological	4 (7)
APACHE II score, median (IQR)	26 (22–30)
SAPS III score, median (IQR)	48 (40–53)
Sepsis/septic shock (Sepsis 3), no. (%)	42 (72)
Mechanical ventilation, no. (%)	44 (76)
Delirium, no. (%)	17 (29)
Duration of ICU stay, days, median (IQR)	4.45 (2–8.5)

Level of education, according to the Swedish national school system (primary—ages 6–15, secondary—ages 15–18, tertiary—university level)

*IQR* interquartile range, *APACHE* Acute Physiology and Chronic Health Evaluation, *SAPS* Simplified Acute Physiology Score

### Changes over time in subjective and objective cognitive function and in psychological distress

There were no significant changes in mean CFQ scores neither between 3 and 6 months nor between 3 and 12 months (Table 3) after ICU discharge.

**Table 3:** Mean scores during follow-up

	Follow-up cohort at 3 months			Follow-up cohort at 6 months				Follow-up cohort at 12 months			
	*N*	Mean scores (SD)	95% CI	*N*	Mean scores (SD)	95% CI	*p* value (∆3 to 6 months)	*N*	Mean scores (SD)	95% CI	*p* value (∆3 to 12 months)
CFQ	40	29.7 (13.7)	25.4–33.9	47	30.3 (14.6)	26.2–34.5	0.62	41	31.6 (13.9)	27.3–35.8	0.18
HADS—anxiety	57	4.2 (4.1)	3.2–5.3	51	3.8 (3.9)	2.7–4.9	0.22	41	3.7 (3.6)	2.6–4.8	0.12
HADS—depression	57	4.7 (4.1)	3.7–5.8	51	4.3 (4.0)	3.3–5.4	0.32	41	4.5 (3.7)	3.4–5.7	0.65
PTSS-10	57	21.2 (11.0)	18.3–24.0	51	22.0 (10.6)	19.1–24.9	0.35	41	21.3 (9.8)	18.3–24.3	0.91
CANTAB											
PRM—% correct	58	82.8 (10.4)	80.1–85.5	51	85.9 (10.3)	83.8–88.7	0.03	45	85.2 (10.1)	82.2–88.1	0.12
RVP—RVP A’	57	0.87 (0.06)	0.85–0.88	47	0.89 (0.06)	0.87–0.91	0.01	41	0.90 (0.06)	0.88–0.92	< 0.001
SOC—minimum moves	57	8.4 (1.9)	7.9–8.9	49	8.8 (1.9)	8.3–9.3	0.19	45	9.4 (1.9)	8.9–10.0	< 0.001
SSP—span length	58	5.8 (1.3)	5.5–6.2	51	5.9 (1.3)	5.6–6.3	0.60	45	5.9 (1.3)	5.6–6.3	0.61

Results shown are mean scores at all time points for each individual self-rating questionnaire and four of the outcome measures from the conducted CANTAB cognitive tests

*CFQ* Cognitive Failures Questionnaire, *HADS* Hospital Anxiety and Depression Scale, *PTSS-10* Post-Traumatic Symptoms Scale, *CANTAB* Cambridge Neuropsychological Test Automated Battery, *SD* standard deviation, *CI* confidence interval, *N* number of patients that completed each questionnaire/test at that time point

At 3 months, 34% (*N* = 20) of the included patients suffered from cognitive impairment according to the criteria [25]. At 6 months, 18% (*N* = 9) of patients met the criteria, and 16% (*N* = 7) at 12 months. Mean scores of the CANTAB tests at each of the follow-up time points are presented in Table 3. There was a significant difference in mean scores between follow-up at 3 and 6 months in two of the four outcome measures, i.e., the RPV A’ score and the PRM—% correct score. Between 3 and 12 months, the mean RPV A’ score and the SOC—problems with min moves improved significantly. However, the SSP—span length did not change significantly between 3, 6, and 12 months Mean scores for HADS—anxiety, HADS—depression, and PTSS-10 are presented in Table 3, and there was no significant difference during the follow-up period.

HADS scores > 10 [31] and PTSS-10 score > 35 [32] were considered indicative of clinically relevant symptoms. At 3 months, 9% of respondents scored > 10 in HADS—anxiety. At 6 and 12 months, 10% and 2% of patients scored > 10 in HADS—anxiety. In HADS—depression, 12% of respondents scored > 10 at 3 months. At 6 and 12 months, 10% of patients scored > 10 respectively. We observed at 3 months that 11% of patients scored above 35 in PTSS-10. At 6 months, the number > 35 was 6%, and at 12 months 5% (Table 4).

**Table 4:** Fractions of patients scoring above clinical cut-offs at follow-up

	Patients scoring above clinical cut-off (HADS, PTSS-10) or with scores equating to cognitive impairment (CANTAB)		
	Follow-up at 3 months	Follow-up at 6 months	Follow-up at 12 months
HADS, no. (%)			
Anxiety	5 (9)	5 (10)	1 (2)
Depression	7 (12)	5 (10)	4 (10)
PTSS-10, no. (%)	6 (11)	4 (6)	2 (5)
CANTAB, no. (%)	20 (34)	9 (18)	7 (16)

Patients were divided into cases and non-cases based on validated clinical cut-offs (HADS and PTSS-10) or by meeting criteria for objective cognitive impairment based on CANTAB scores. Objective cognitive impairment was defined as a score above − 1.5 standard deviations from the norm in three or more outcome measures or scoring above − 2.0 standard deviations from the norm in two or more outcome measures. Objective cognitive impairment (CANTAB) decreased in the cohort over time

*HADS* Hospital Anxiety and Depression Scale, *PTSS-10* Post-Traumatic Symptoms Scale, *CANTAB* Cambridge Neuropsychological Test Automated Battery

### Correlations between subjective and objective cognitive function

**Fig. 2:**
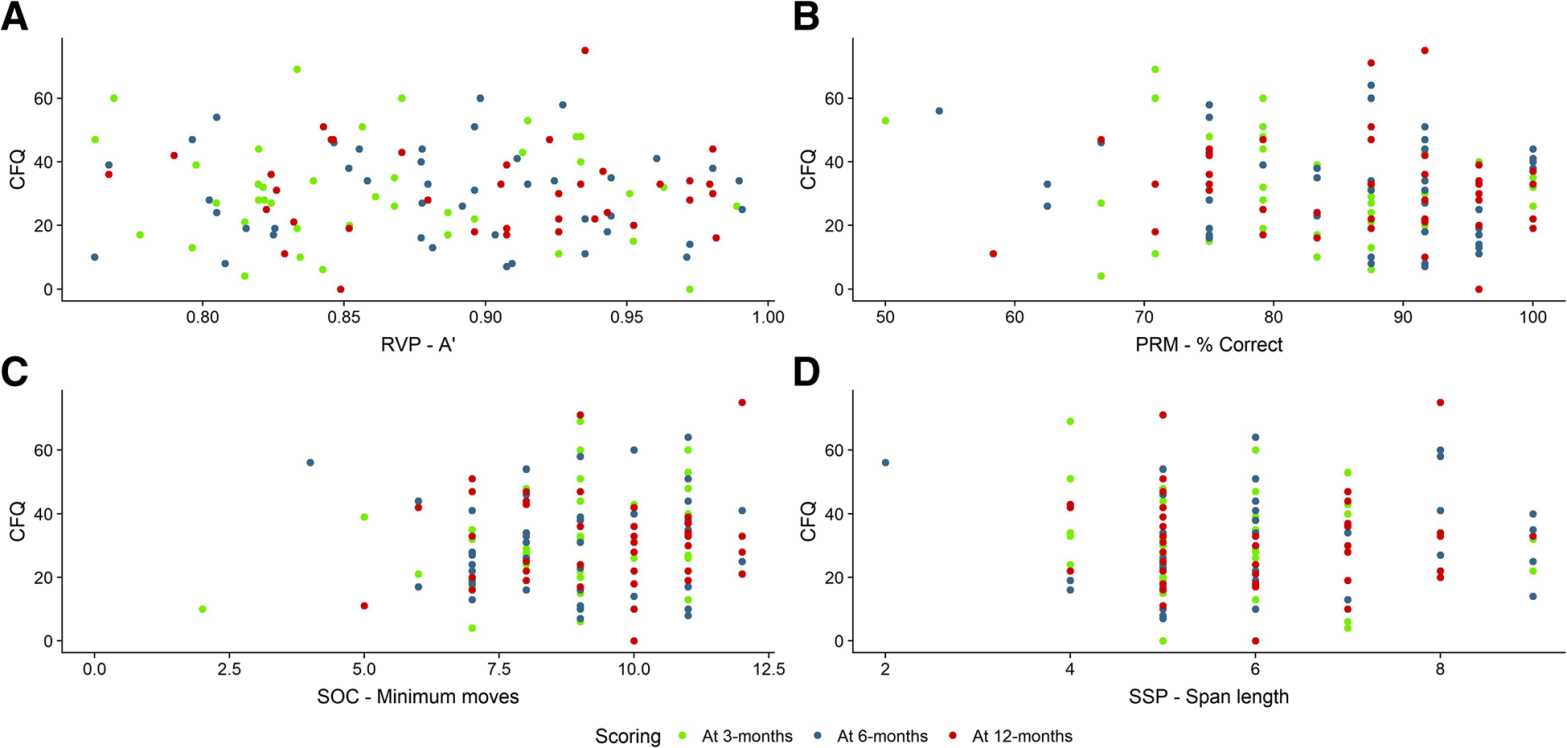
Correlation between subjective and objective cognitive function. Spearman’s rank correlation coefficient was calculated for CFQ and four of the CANTAB outcome measures at all time points. **a** Plots of CFQ scores and % correct on the PRM test; *r* = − 0.134, *p* = 0.408, 95% CI [− 0.428, 0.185] at 3 months; *r* = − 0.106, *p* = 0.483, 95% CI [− 0.384, 0.190] at 6 months; *r* = − 0.070, *p* = 0.664, 95% CI [− 0.370, 0.243] at 12 months. **b** Plots of CFQ scores and the outcome measure RVP A’; *r* = − 0.018, *p* = 0.915, 95% CI [− 0.331, 0.300] at 3 months; *r* = − 0.084, *p* = 0.596, 95% CI [− 0.378, 0.226] at 6 months; *r* = − 0.067, *p* = 0.695, 95% CI [− 0.382, 0.263] at 12 months. **c** Plots of CFQ scores and number of tests with minimum moves completed in the SOC test; *r* = 0.207, *p* = 0.200, 95% CI [− 0.112, 0.487] at 3 months; *r* = 0.066, *p* = 0.667, 95% CI [− 0.232, 0.353] at 6 months; *r* = − 0.028, *p* = 0.864, 95% CI [− 0.332, 0.282] at 12 months. **d** Plots of CFQ scores and span length achieved on the SSP test; *r* = − 0.023, *p* = 0.890, 95% CI [− 0.332, 0.291] at 3 months; *r* = 0.257, *p* = 0.085, 95% CI [− 0.036, 0.509] at 6 months; *r* = − 0.109, *p* = 0.498, 95% CI [− 0.403, 0.206] at 12 months. No statistical significance was reached. CFQ, Cognitive Failures Questionnaire; CANTAB, Cambridge Neuropsychological Test Automated Battery; PRM, pattern recognition memory; RVP, rapid visual information processing; SOC, Stockings of Cambridge; SSP, spatial span; CI, confidence interval

The correlations between CFQ scores and the outcome measures from CANTAB (PRM—% correct, RPV A’, SOC—problems with min moves, SSP—span length) were between *r* = −0.208–0.257 and *p* = 0.085–0.915, and none reached statistical significance (Fig. 2).

### Correlations between cognitive function and psychological distress

**Fig. 3:**
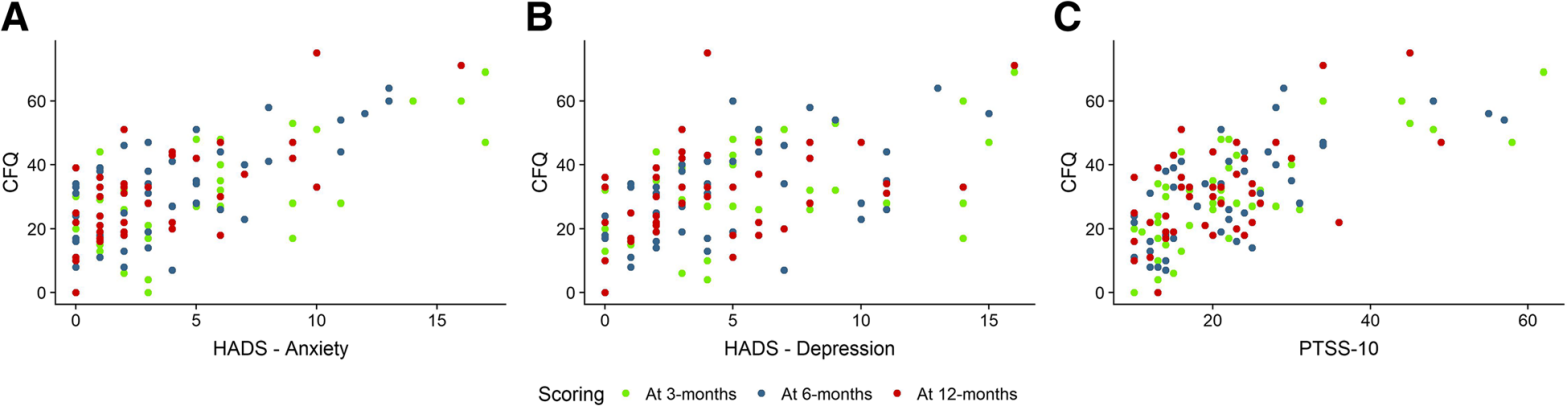
Correlation between subjective cognitive function and psychological distress. Spearman’s rank correlation coefficient was used to establish statistical significance. **a** Plots of CFQ scores against the HADS anxiety subscale (*r* = 0.550 at 3 months, *p* ≤ 0.001; *r* = 0.645 at 6 months, *p* < 0.001; *r* = 0.552 at 12 months, *p* < 0.001). **b** Plots of CFQ scores against the HADS depression subscale (*r* = 0.510 at 3 months, *p* < 0.001; *r* = 0.590 at 6 months, *p* < 0.001; *r* = 0.372 at 12 months, *p* = 0.023). **c** Plots of CFQ scores against the PTSS-10 score (*r* = 0.710 at 3 months, *p* ≤ 0.001; *r* = 0.710 at 6 months, *p* ≤ 0.001; *r* = 0.440 at 12 months, *p* ≤ 0.01). CFQ, The Cognitive Failures Questionnaire; HADS, Hospital Anxiety and Depression Scale; PTSS-10, Post-Traumatic Symptom Scale

We found significant positive correlations between the CFQ score and the HADS—anxiety score, HADS—depression score, and PTSS-10 score at 3-month and 6-month follow-up (Fig. 3). Further, there was a significant correlation between the CFQ and the HADS—anxiety score at 12 months (Fig. 3a), while the correlation between CFQ and HADS—depression score and PTSS-10 score was weaker at this time point (Fig. 3b, c). No significant correlation was observed between the CANTAB outcome measures and the HADS scores and PTSS-10 scores, except between the PRM—% correct score and the HADS score and PTSS-10 score at 3 months, which showed a weak but significant correlation (Table 5).

**Table 5:** Objectively measured cognitive performance outcome measures from CANTAB against self-rating scores for anxiety and depression (HADS) and PTSD (PTSS-10) using Spearman’s rank correlation

	PRM—% correct		RVP A’		SOC—problems with min moves		SSP—span length	
	*r*	*p*	*r*	*p*	*r*	*p*	*r*	*p*
HADS—anxiety								
At 3 months	− 0.319	0.015	− 0.041	0.765	0.056	0.682	0.172	0.202
At 6 months	− 0.054	0.708	0.102	0.494	− 0.059	0.687	0.199	0.161
At 12 months	− 0.161	0.315	0.064	0.707	0.037	0.817	− 0.191	0.231
HADS—depression								
At 3 months	− 0.348	0.008	− 0.070	0.610	0.029	0.835	0.085	0.529
At 6 months	− 0.145	0.310	− 0.088	0.557	− 0.024	0.868	0.028	0.843
At 12 months	− 0.210	0.188	− 0.113	0.507	0.037	0.819	− 0.281	0.075
PTSS-10								
At 3 months	− 0.329	0.013	− 0.070	0.609	0.107	0.434	0.136	0.312
At 6 months	− 0.170	0.234	− 0.028	0.852	0.043	0.770	0.102	0.476
At 12 months	− 0.129	0.423	0.044	0.795	0.154	0.336	− 0.113	0.481

*PRM* pattern recognition memory—% correct, *RVP* rapid visual information processing—RVP A’, *SOC* Stockings of Cambridge—problems solved with minimum moves, *SSP* spatial span—span length, *HADS* Hospital Anxiety and Depression Scale, *PTSS-10* Post-Traumatic Stress Symptoms Scale-10

## Discussion

In this prospective cohort study of ICU survivors, we observed a lack of clinically relevant correlation between subjective and objective cognitive function test results. Psychological distress correlated strongly to subjective cognitive function, as previously reported [12], whereas there was a weak association between psychological symptoms and objectively assessed cognitive function. To the best of our knowledge, this study is the first to examine the association between subjective and objective cognitive function in ICU survivors.

Cognitive performance after intensive care, as a facet of PICS, has become an established area of research. With growing awareness of PICS, there is a need for screening tools in clinical practice to identify cognitive impairment in ICU survivors. Broad availability of such tools would enable convenient assessment of cognitive function at follow-up in outpatient clinics and help identify patients in need of additional evaluation, training, and support. Assessment of cognitive function is complicated by that patients are often biased in their ability to self-evaluate [16] and self-rating questionnaires may be of limited value as screening tools for cognitive dysfunction.

CFQ has been used as a measure of cognitive function in ICU survivors [14, 15], which has led to the suggestion to use CFQ as a screening tool to select patients for further objective cognitive evaluation [33]. However, the observations in the present study on the correlation of subjective CFQ scores and objective CANTAB results indicate a lack of clinically relevant correlation in ICU survivors. These findings support the important notion that subjective and objective tests measure different aspects of cognitive function. Although both may be relevant, they may not be interchangeable.

Discrepancies between reported subjective levels of function and objectively tested function are not uncommon in other conditions. For example, fatigue is a highly disabling symptom and is common in various medical conditions [34, 35], including in ICU survivors [36]. Fatigue can be measured subjectively (using self-rating questionnaires) or objectively (using long or intense physical or mental tasks). However, the objective measures of fatigue do not necessarily relate to the subjective measures [37]. This indicates that subjective and objective measures of fatigue (and cognitive function) probably assess distinct components. Subjective and objective measurements of fatigue are both clinically relevant, as subjective feelings of fatigue affect patients’ quality of life [38] as much as objective difficulties to perform physical or mental tasks [39]. The same applies to cognitive function. While a patient may subjectively experience increased cognitive failures in their everyday life, this does not necessarily correlate to objectively tested cognitive performance in a controlled setting. In other words, patients with good performance in the conventional testing environment could still have problems in their daily life, which might be better reflected in subjective scoring. Conversely, good subjective scoring does not rule out objective cognitive impairment.

Cognitive impairment after critical illness is often reduced over time [7]. Interestingly, mean CFQ scores did not improve over time in this cohort, whereas objective cognitive function measured by sustained attention (RVP) and executive function (SOC) did. This might indicate that a proportion of ICU survivors have difficulties in estimating their own cognitive function. For example, individuals suffering from severe cognitive impairment may not be able to provide a veridical judgment of their cognitive function and overestimate their performance. This would be in line with the theoretical explanation of the age-CFQ paradox (in which older people that on average have reduced cognitive capacity rate themselves with moderate to good scores on the CFQ). Increasing forgetfulness may approach a point at which one forgets, that which has been forgotten [40]. This is an inherent methodological problem with subjective cognitive evaluation, which may lead to that those much worse off might be misclassified with cognitive self-rating screening tools, a phenomenon that might weaken the correlation between subjective and objective measurements of cognitive function.

The cognitive domains reported to be mostly affected after intensive care involve memory, attention, processing speed, visuospatial ability, and executive function [11]. CANTAB tests in the present study were accordingly chosen to measure these functions. While CFQ and CANTAB both assess memory and executive function, the two tests measure different functions to some extent. Importantly, the aim of this study was not to assess whether CFQ is adequate for assessment of the selected CANTAB tests. Rather, we set out to determine if the CFQ score reflects the impairments that have been objectively identified in ICU survivors.

The finding of a strong correlation between psychological symptoms and subjective cognitive function agrees with previous studies [12] and is also in line with the known cognitive effects of psychological distress. Indirectly, this correlation suggests that subjective cognitive performance is a relevant patient outcome.

The main question in this study was whether a test of subjective cognitive function can replace objectively assessed cognitive function and be a relevant and useful tool in ICU follow-up. The results of the study raise a new question: What is the most relevant outcome measure in ICU survivors—the patient’s perception of their cognitive function or their objectively tested cognitive function? The purpose of ICU follow-up is to identify patients at risk and those that can benefit from aid and interventions. Both subjective and objective cognitive impairment are relevant, because aspects of these functions are important in different facets of people’s lives, including patients’ quality of life and ability to work. Accordingly, the answer likely depends on the purpose of the cognitive evaluation.

## Limitations

The nature of ICU admission precludes pre-admission measurement of individual baseline cognitive function, and reference values must therefore be derived from the general population. This limitation does not however impact the study of correlation between different measures of cognitive function in individual patients.

To partly mitigate the challenge with the lack of data on the pre-admission cognitive function, inclusion criteria were designed to strictly select patients without known previous cognitive dysfunction or mental illness. This to identify ICU stay-related onset of cognitive impairment as a part of PICS. The narrow inclusion and exclusion criteria limited the number of individuals that were enrolled during the study period, and this study was not powered to detect weak correlations. Importantly, statistical analysis of the data indicated that the precision of the estimates in the study is sufficient to exclude substantial correlation. This observation along with the scatterplots shown in Fig. 2 lends support to our conclusion that a substantial correlation between CFQ and the four outcome measures of CANTAB is lacking in this study. Of note, the central question here was whether subjective cognitive function as measured by CFQ reflects objective cognitive function as measured by CANTAB to a degree that makes CFQ useful as a clinical screening instrument for impairment of objective cognitive function in ICU survivors.

Some of the ICU survivors had not regained their pre-admission level of function at the follow-up visits. To avoid patient dropout, the formal testing in this study was limited to 30 min. It cannot be excluded that individuals’ personal motivation to participate may have varied between test occasions. Moreover, fatigue was not measured, and it is possible that the level of fatigue influenced the cognitive performance as reflected by CANTAB scores. Finally, testing took place in a hospital office environment at daytime, which might not reveal mild cognitive problems that may show diurnal variation and become more pronounced in states of physical fatigue.

## Conclusion

This prospective follow-up study of ICU survivors showed a lack of clinically relevant correlation between subjective and objective cognitive function as measured with CFQ and CANTAB, while subjective cognitive function correlated significantly with psychological symptoms throughout the 12-month follow-up period. The findings highlight the complexity of cognitive function testing in ICU survivors. Further initiatives to validate effective screening methods of subjective and objective cognitive problems in ICU survivors are needed, since these problems are substantial in ICU survivors and can affect the patients’ recovery for years after intensive care.

## Data Availability

The datasets used and analyzed during the current study are available from the corresponding author on reasonable request.

## Abbreviations

APACHE II: Acute Physiology and Chronic Health Evaluation
CANTAB: Cambridge Neuropsychological Test Automated Battery
CFQ: Cognitive Failures Questionnaire
CI: Confidence interval
ECMO: Extracorporeal membrane oxygenation
HADS: Hospital Anxiety and Depression Scale
ICU: Intensive care unit
IQR: Interquartile range
PICS: Post-intensive care syndrome
PRM: Pattern recognition memory
PTSD: Post-traumatic stress disorder
PTSS-10: Post-Traumatic Stress Symptoms Scale 10
RVP: Rapid visual information processing
SAPS III: Simplified Acute Physiology Score
SOC: Stockings of Cambridge
SSP: Spatial span
